# Rechallenge With Cisplatin After an Acute Coronary Event

**DOI:** 10.1016/j.jaccas.2025.106591

**Published:** 2026-01-21

**Authors:** Zehra Jaffery, Rick L. Jobski, Richard S. Siegel, Anju Nohria

**Affiliations:** a Department of Cardiology, Northwest Community Hospital, Arlington Heights, Illinois, USA; b Illinois Cancer Specialists, Arlington Heights, Illinois, USA; c Division of Cardiovascular Medicine, Brigham and Women's Hospital, Boston, Massachusetts, USA

**Keywords:** acute coronary syndrome, cisplatin, rechallenge

## Abstract

**Background:**

Testicular cancer is the most common malignancy in young men, and cisplatin-based chemotherapy provides excellent long-term survival. However, cisplatin-associated cardiovascular toxicity spans a broad spectrum, and evidence guiding rechallenge after an acute coronary event is limited.

**Case Summary:**

A 24-year-old man with testicular cancer and no cardiovascular risk factors developed chest pain and elevated troponins early in his cisplatin therapy. Coronary angiography revealed a nonocclusive left anterior descending artery thrombus. After treatment with antiplatelet and anticoagulant therapy, the thrombus resolved, and he subsequently underwent a carefully monitored cisplatin rechallenge.

**Discussion:**

Given cisplatin's curative benefit, a multidisciplinary team—including cardiology, oncology, and pharmacy—assessed the risks and benefits of continuing therapy. With close surveillance and optimized medical management, including anti-ischemic therapy and anticoagulation, cisplatin was safely reintroduced.

**Take-Home Messages:**

Cisplatin-induced myocardial infarction requires prompt recognition and coordinated care. With multidisciplinary evaluation, rechallenge can be safely performed in select patients.

## Central Illustration

**Figure:**
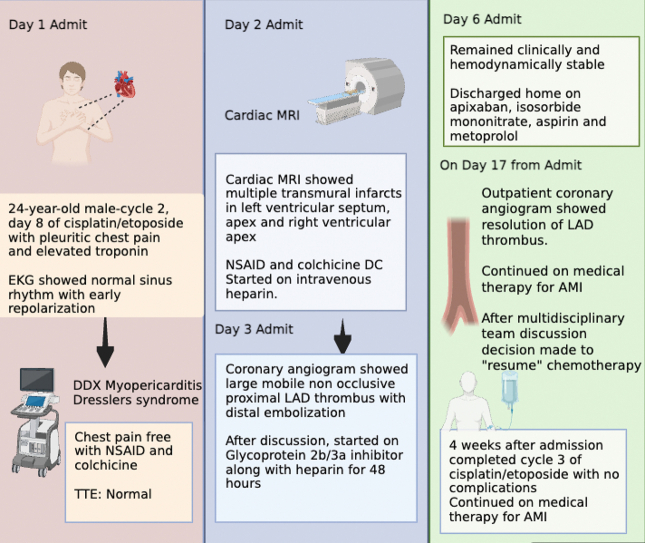

**Visual Summary:**
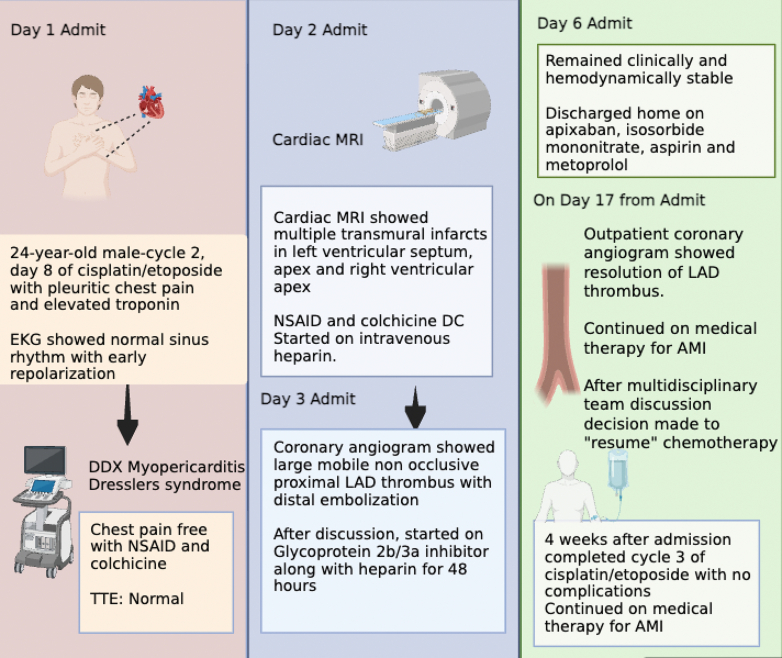
Patient Presentation and Treatment Course The figure was created using BioRender. AMI = acute myocardial infarction; DC = discontinuation; DDX = differential diagnosis; EKG = electrocardiogram; LAD = left anterior descending artery; MRI = magnetic resonance imaging; NSAID = nonsteroidal anti-inflammatory drug; TTE = transthoracic echocardiogram.

## History of Present Illness

A 24-year-old man with testicular cancer presented to the emergency department on day 8 of cycle 2 of cisplatin and etoposide chemotherapy with 24 hours of pleuritic chest pain. On examination, he was hemodynamically stable with an unremarkable physical examination. Electrocardiogram demonstrated normal sinus rhythm with early repolarization. High-sensitivity cardiac troponin was elevated at 8,100 ng/L, with subsequent serial measurements of 6,422, 10,481, and 9,067 ng/L.

**Take-Home Messages**

• Cisplatin-induced myocardial infarction requires prompt recognition and coordinated care.
• With multidisciplinary evaluation, rechallenge can be safely performed in select patients.

## Past Medical History

He was recently diagnosed with a stage IIB (T2b N1 M0) nonseminomatous germ cell tumor of the testis and had undergone left orchiectomy 2 months earlier. His planned chemotherapy regimen consisted of 4 cycles of cisplatin/etoposide because he opted to avoid bleomycin because of its potential for pulmonary toxicity. He had a body mass index of 33 kg/m^2^ and no other traditional cardiovascular risk factors.

## Differential Diagnosis

In the context of pleuritic chest pain with an elevated troponin, the differential diagnosis was myopericarditis and cisplatin-induced coronary vasospasm with Dressler syndrome.

## Investigations

**Figure 1:**
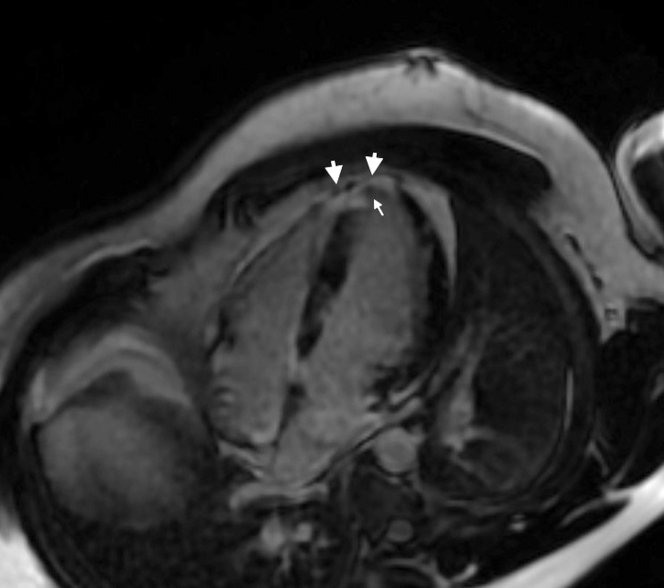
Cardiac Magnetic Resonance Four-chamber view showing delayed contrast enhancement in the left ventricular septum, apex, and right ventricular apex (large arrows). Small arrow displays microvascular obstruction.

Transthoracic echocardiography revealed normal cardiac structure and function with no pericardial effusion. As part of the diagnostic work-up, cardiac magnetic resonance was performed. Cardiac magnetic resonance demonstrated a normal left ventricular ejection fraction of 79% with a small area of apical hypokinesis. There was transmural late gadolinium enhancement involving approximately 15% of the myocardium with associated edema affecting the anteroseptum, apex, and right ventricular apex (Figure 1).

**Figure 2:**
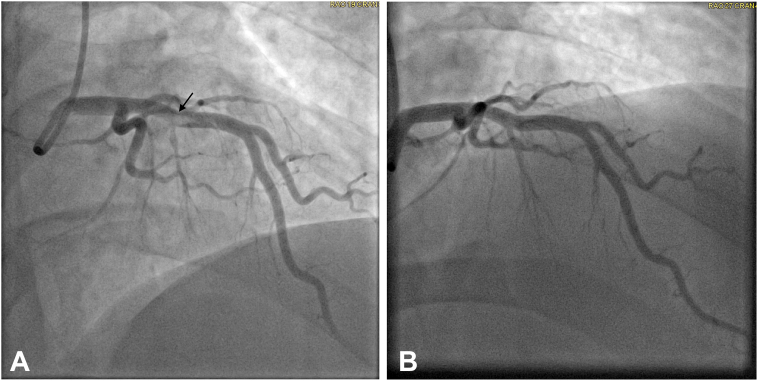
Coronary Angiograms (A) Initial coronary angiogram with a large filling defect (thrombus) in the proximal left anterior descending (LAD) artery (arrow). (B) Two-week coronary angiogram with resolution of the thrombus in the proximal LAD after medical management.

Given the known association between cisplatin and arterial thrombosis, coronary angiography was performed. This revealed a large, nonocclusive, mobile thrombus in the proximal left anterior descending (LAD) artery at the level of a large septal perforator, with poor distal runoff in the distal LAD, likely due to distal embolization (Figure 2A).

## Management

On admission, he was started on colchicine and nonsteroidal anti-inflammatory drugs and was chest pain free within 12 hours. After coronary angiography and multidisciplinary discussion, thrombectomy was deferred because of the high risk of further embolization. He was started on a glycoprotein 2b/3a inhibitor along with heparin for 48 hours. Because he remained asymptomatic, a “watch and wait” approach with systemic anticoagulation and planned follow-up coronary angiography to reassess the thrombus was deemed the safest strategy. A bubble study was negative, and further work-up showed no evidence of venous thrombosis. He was discharged on hospital day 5 on metoprolol, aspirin, isosorbide mononitrate, and apixaban. A repeat coronary angiogram performed 2 weeks later demonstrated complete resolution of the thrombus with normal coronary arteries (Figure 2B).

There was discussion between the oncology and cardio-oncology teams regarding the safety of resuming cisplatin given his recent acute myocardial infarction (AMI) and his poor long-term cancer prognosis with cessation of therapy. Based on review of published case series, his young age, being symptom-free, and his stable clinical status on current medical treatment and cancer prognosis, the risk-benefit ratio was thought to favor resumption of his chemotherapy regimen with close cardiac monitoring.

## Outcomes and Follow-Up

He completed cycle 3 without complications but opted to forgo cycle 4 in favor of active surveillance. Aspirin therapy was continued for 1 year in accordance with AMI guidelines. At his 6-month follow-up, he requested discontinuation of isosorbide mononitrate. At 2-year follow-up, he remains free of recurrent cardiovascular events, his tumor is in complete remission, and he remains on metoprolol and apixaban. The optimal duration of antithrombotic therapy in this context is unclear. Given the lack of evidence supporting long-term use, we plan to reassess this at his 3-year follow-up. If antithrombotic therapy is discontinued at that time, low-dose aspirin will be restarted and continued indefinitely. Metoprolol is planned for at least 3 years after his cardiac event and will also be readdressed at this next visit. His traditional cardiovascular risk factors will require closely monitoring and treatment going forward.

## Discussion

We describe the case of a 24-year-old man who developed an AMI from a large, mobile LAD thrombus during cisplatin therapy for testicular cancer. The event was successfully managed medically, and he was later safely rechallenged with cisplatin without additional cardiovascular complications.

The risk of cisplatin-induced cardiovascular disease is greatest within the first year after treatment but may persist or increase years later, suggesting a biphasic pattern of risk.^1^ Thromboembolic complications occur in approximately 9% to 11%^2^ of patients immediately after cisplatin-based chemotherapy, and chest pain is reported in up to 40%.^3^ Coronary angiography may reveal single and even multivessel coronary thrombosis without underlying atherosclerosis.^4^ Experimental evidence supports plaque erosion as the leading mechanism, with cisplatin inducing endothelial damage, thromboxane production, and platelet activation and aggregation. Vasospastic angina has also been reported. This early vascular toxicity is pathologically distinct from chronic atherosclerotic disease. Notably, cisplatin levels can remain detectable in the body for years after therapy, maintaining a prolonged risk of chest pain and acute ischemic events.^5^

Published literature regarding continuation of cisplatin after an acute cardiovascular event is limited to conflicting case reports. Morrow et al^6^ described a 45-year-old with an AMI and cardiac arrest treated with primary percutaneous intervention, attributed to underlying atherosclerosis. He completed chemotherapy on standard post-myocardial Infarction (MI) therapy but experienced another AMI 10 months later due to progressive atherosclerosis and left ventricular thrombus, prompting anticoagulation. Scafa-Udriste et al^7^ reported a case of a 34-year-old man with an acute inferior MI and mild right coronary artery stenosis who continued cisplatin after initiation of standard secondary prevention for MI. In contrast, our patient who was younger and had no evidence of atherosclerosis, presented with a large mobile LAD thrombus and multiple infarcts, and was maintained on long-term anticoagulation to prevent recurrent events.

A “high-risk vascular fingerprint” has recently been proposed for patients with testicular cancer receiving cisplatin, defined by the presence of ≥3 of the following: body mass index ≥25 kg/m^2^, current smoking, hypertension (blood pressure >140/90 mm Hg or treated), hyperlipidemia (or treated), and elevated fasting plasma glucose.^8^ Our patient did not meet these high-risk criteria on admission.

The incidence of testicular cancer in young men has been rising, and although cure rates are excellent, evidence guiding cisplatin continuation after an AMI remains limited. Moreover, there are no data defining optimal cardioprotection strategies in this population. Establishing national registries to track patients who experience acute cardiovascular events during or after cisplatin treatment could provide valuable insights to inform management.

## Conclusions

Cisplatin-associated cardiovascular events, although uncommon, can be life-threatening. Our patient presented with Dressler syndrome and was found to have a large, mobile LAD thrombus. Early recognition and prompt management were lifesaving, and a multidisciplinary approach enabled continuation of his chemotherapy. He remains on long-term anticoagulation and has remained free of recurrent events 2 years after his initial presentation.

## Funding Support and Author Disclosures

Dr Nohria has received unrelated research support from Bristol Myers Squibb; consulting fees from AstraZeneca and Takeda Oncology; speaker fees from MLI Education; and is a paid clinical expert for Teledoc. All other authors have reported that they have no relationships relevant to the contents of this paper to disclose.
